# Anticoagulation strategies in COVID-19 infected patients receiving ECMO support

**DOI:** 10.1051/ject/2023027

**Published:** 2023-09-08

**Authors:** Dayne Diaz, Jenny Martinez, Grant Bushman, William R. Wolowich

**Affiliations:** 1 Department of Pharmacy, Mount Sinai Medical Center, Pharmacy Suite 2020 4300 Alton Rd. Miami Beach FL 33140 United States; 2 Department of Pharmacy Practice, Nova Southeastern University 3300 S University Dr Fort Lauderdale FL 33328 United States

**Keywords:** ECMO, COVID, Anticoagulation, Bivalirudin, Heparin

## Abstract

*Background*: Hospitalized COVID-19 patients with hypoxemic respiratory failure may deteriorate despite invasive mechanical ventilation and thus require extracorporeal membrane oxygenation (ECMO) support. Unfractionated heparin (UFH) is the antithrombotic of choice, however, bivalirudin may offer more predictable pharmacokinetics resulting in consistent anticoagulant effects with lower bleeding and thrombotic occurrences. The aim of this study was to evaluate efficacy and safety outcomes in patients undergoing venovenous (VV) ECMO receiving bivalirudin or UFH-based anticoagulation. *Methods*: This retrospective, single-center, observational cohort study included patients with confirmed COVID-19 infection requiring VV ECMO support receiving anticoagulation with UFH or bivalirudin. Primary endpoints were time to reach therapeutic aPTT, percent time spent in aPTT range, and the occurrence of thrombotic events over the entire course of ECMO support. Secondary endpoints included the incidence of major/minor bleeding, the ability to wean off ECMO support, in-hospital mortality, and length of stay. *Results*: Twenty-two patients were included in the study (*n* = 10 UFH, *n* = 12 bivalirudin). Time to therapeutic aPTT was achieved faster with UFH (10 h vs. 20 h). The percentage time spent within the goal aPTT range was similar between UFH and bivalirudin (50% vs. 52%). Thrombotic events were significantly higher in the UFH group (40% DVT, 40% PE, 80% oxygenator thrombus in ECMO machine, 10% ischemic stroke) versus bivalirudin (8% DVT, 17% PE, 33% oxygenator thrombus, no ischemic strokes) (CI 95%, *p* = 0.04). The overall bleeding incidence was higher in the UFH arm (90% vs. 75%). The mortality rate was 90% in the UFH group and 58% in the bivalirudin group. The length of stay was similar between the two study arms. *Conclusion*: In hospitalized patients with COVID-19-associated acute respiratory distress syndrome (ARDS) on VV ECMO support, the use of bivalirudin showed to be a viable anticoagulation alternative in terms of efficacy compared to UFH and resulted in a favorable safety profile with lower rates of bleeding and thrombotic events.

## Introduction

Severe acute respiratory syndrome coronavirus 2 (SARS-CoV-2), the causative virus of coronavirus disease 2019 (COVID-19), was first reported in December 2019 in Wuhan, China. Since then, more than 281 million confirmed cases and 5 million deaths have been reported worldwide [1, 2]. The mortality rate in COVID-19-associated acute respiratory distress syndrome (ARDS) is 45% and the incidence of ARDS among non-survivors of COVID-19 is 90% [3]. Hospitalized patients infected with COVID-19 present with unique progressive hypoxic dyspnea, inflammatory cytokine storm, and hypercoagulability states that are in some instances refractory to conventional therapy.

The indications for the use of Extra Corporeal Membrane Oxygenation (ECMO) have expanded in recent years. ECMO is now an essential step in the treatment of patients who present with pulmonary failure refractory to conventional management. ECMO support is initiated in cases of refractory hypoxemia unresponsive to traditional care such as high positive end-expiratory pressure (PEEP) strategy, recruitment maneuvers, prone positioning, antivirals, as well as optimized analgesia, sedation, and neuromuscular blockade. Most COVID-19 patients (>90%) requiring ECMO for ARDS have been supported using venovenous (VV) ECMO [4]. The World Health Organization (WHO) publication of August 2020 recommends the use of ECMO for the treatment of COVID-19-associated with ARDS to be offered only in expert centers with sufficient experience in the management of ECMO support [5]. Recent studies have shown, in patients with COVID-19 supported with ECMO, both the estimated mortality 90 days after ECMO initiation and the mortality in patients who achieved a final disposition of death or discharge, to be less than 40% [6].

Systemic anticoagulation in ECMO becomes an imperative necessity due to the hypercoagulable state caused by COVID-19 disease [7]. Anticoagulation is needed to reduce the occurrence of thromboembolic complications in the patient and in the ECMO circuit. To date, unfractionated heparin (UFH) is the primary antithrombotic cited in the extracorporeal life support organization (ELSO) guidelines [8] and it is frequently used during ECMO support. UFH has been the most studied anticoagulant in this patient population and has the advantage of a pharmacologic reversal agent, protamine. However, direct thrombin inhibitors (DTIs) such as bivalirudin may offer more predictable pharmacokinetics resulting in consistent anticoagulant effects with lower bleeding, thrombotic occurrences, and no risk for heparin-induced thrombocytopenia (HIT).

Bivalirudin directly binds to the active sites of thrombin providing a steadier pharmacokinetics profile and a greater reduction in thrombin when compared to UFH [9]. Bivalirudin, in contrast to UFH, has the ability to bind both circulating and clot-bound thrombin independent of antithrombin activity [10]. Additionally, bivalirudin possesses a short half-life of 25 min and a quick offset of action, which allows rapid titration to achieve desired anticoagulation levels as well as rapid cessation of anticoagulant effects when needed. To date, however, there is no specific reversal agent available for bivalirudin.

Recent literature has shown goal-activated partial thromboplastin time (aPTT) values are reached more consistently in the COVID-19 population supported with VV ECMO with little to no increase in bleeding or thrombotic events when using bivalirudin for anticoagulation [11–13]. Although there is preliminary literature about the use of bivalirudin for anticoagulation during ECMO, the published data and protocols regarding its dosing and monitoring are limited in patients with COVID-19 dependent on ECMO support.

This study aimed to evaluate efficacy and safety outcomes in hospitalized patients with COVID-19 undergoing VV ECMO receiving bivalirudin and UFH-based anticoagulation.

## Materials and methods

### Study design and subjects

This was a retrospective, single-center, observational cohort study conducted at Mount Sinai Medical Center (MSMC) in Miami Beach, Florida. The research was approved by the MSMC Investigational Review Board on October 6th, 2021. Hospitalized adult patients with confirmed COVID-19 infection requiring VV ECMO support who received anticoagulation with UFH or bivalirudin between February 2020 and November 2021 were identified from MSMC electronic health records (EHR) and included for analysis. Data manually extracted from the EHR included demographic characteristics, VV ECMO indication, cannulation sites and duration, bivalirudin or UFH dosing and monitoring, thromboembolic and bleeding incidences, as well as length of stay and in-hospital mortality.

Eligible patients were 18 years of age or older with a confirmed COVID-19 diagnosis who met the criteria for VV ECMO support due to respiratory failure and were on systemic anticoagulation with bivalirudin or UFH. Patients younger than 18 years of age or older than 65 years of age, with a history of severe coagulopathy, or on VA ECMO were excluded from the group analysis.

Primary endpoints were time to reach therapeutic activated partial thromboplastin time (aPTT), percent time spent in aPTT therapeutic range (%TTR), and the occurrence of thrombotic events over the entire course of VV ECMO support. Secondary endpoints assessed the incidence of major and minor bleeding, adverse drug reactions, ability to wean off ECMO support, in-hospital mortality, and length of stay.

### Anticoagulation treatment

Study subjects were on anticoagulation treatment after VV ECMO cannulation with either UFH or bivalirudin.

While on bivalirudin, aPTT values were initially measured every 2–6 h and 2–3 times daily after aPTT therapeutic goal was achieved. Individualized aPTT target goals were determined by the cardiothoracic surgeon. The aPTT target goals were primarily in the range of 60–80 s (1.5–2 times upper limit of normal [ULN]) and adjusted based on patient-specific characteristics and bleeding risk factors. Patients were evaluated on a daily basis to assess for ECMO circuit functioning, bleeding, and thrombus in the patient or oxygenator, as well as hematology and chemistry status.

Bivalirudin was administered to the majority of patients at an initial dose of 0.15 mg/kg/h and titrated based on aPTT target levels. For patients receiving renal replacement therapy, the initial dose was adjusted to begin at 0.03–0.07 mg/kg/h. On average, UFH was started at 10–15 units/kg/h and titrated to achieve target aPTT goals adhering to hospital protocol (Tables 1 and 2).

**Table 1:** Bivalirudin rate adjustments protocol.

Goal aPTT 1.5 – 2 × ULN:
Start infusion at 0.15 mg/kg/h, if on dialysis start at 0.07 mg/kg/h
For aPTT ≤ 20 goal: increase rate by 20% and repeat aPTT in 2 h
For aPTT ≤ 10 goal: increase rate by 10% and repeat aPTT in 2 h
For aPTT at goal: continue current rate and repeat aPTT in 4 h
For aPTT ≥ 10 goal: decrease rate by 10% and repeat aPTT in 2 h
For aPTT ≥ 20 goal: decrease rate by 20% and repeat aPTT in 2 h
For aPTT > 100 for two consecutive levels: HOLD drip for 2 h, contact MD, decrease rate by 20% when resumed, and repeat aPTT in 2 h

**Table 2:** Heparin rate adjustments for maintenance infusion protocol.

Start infusion at 15 units/kg/h (max 1800 units/h)				
Goal aPTT (44–66, 67–92):				
44–66	67–92	Bolus dose	Change current infusion	Order next PTT
<23	<47	Give maintenance bolus dose	Increase rate by 4 units/kg/h	4 h
23–43	47–66	None	Increase rate by 2 units/kg/h	4 h
44–66	67–92	None	Goal range, continue current rate	4 h × 1, then every AM
67–92	93–107	None	**HOLD for 30 min**, then decrease rate by 2 units/kg/h	4 h
93–107	108–126	None	**HOLD for 1 h**, then decrease rate by 3 units/kg/h	4 h after start of infusion
>107	>126	None	**HOLD for 2 h**, then decrease rate by 4 units/kg/h	4 h after start of infusion

**If PTT > 120 for two consecutive PTT values and/or > 200 for any PTT result, HOLD infusion and notify Physician**.

### Study variables and assessments

Data collected by the primary investigators from the EHR was retrospectively extracted for demographics, COVID-19-diagnosis, VV ECMO cannulation sites and dates, aPTT, hematology and chemistry laboratories, bleeding and thrombotic incidence, anticoagulation treatment, and study outcomes. The main outcomes evaluated were time to reach aPTT goal, percentage time in target aPTT, and the occurrence of thrombotic events. Secondary outcomes assessed minor and major bleeding incidence, ability to wean off ECMO support, in-hospital mortality, and overall length of stay.

### Definition of events

Therapeutic aPTT was defined as an aPTT value within the pre-determined range dictated by the cardiothoracic surgeon. Time to therapeutic aPTT was defined as the time in hours it took for each patient to achieve 1.5–2.0 times the patient’s baseline aPTT or predetermined aPTT goal as per the surgeon’s discretion. Percentage time in therapeutic aPTT was calculated as an average of patients’ daily aPTT values within the target range divided by the total number of days on anticoagulation (TTR = [% of time/day patient at therapeutic aPTT/total days under anticoagulation]).

Major bleed, as defined by ELSO guidelines, was classified as a hemoglobin drop of at least 2 g/dL in a 24-h period, transfusion requirement of one or more 10 mL/kg packed red blood cells (PRBC) transfusions over 24 h, or bleeding requiring surgical intervention. Minor bleeding was classified as oropharynx bleed, epistaxis, coffee-ground nasogastric tube (NGT) output, and cannulation site bleed not requiring surgical intervention, blood, platelets, or cryoprecipitate transfusions. Thrombotic events were defined as events requiring VV ECMO suspension or circuit oxygenator exchange, or thromboembolic events detected by duplex ultrasonography or computed tomography (CT). The incidence of thrombotic events was determined by assessing clinical documentation in the patient’s EHR for occurrences of deep vein thrombosis (DVT), pulmonary embolism (PE), circuit clotting necessitating equipment exchange, or cerebral infarction.

### Statistical analysis

Data were analyzed using chi-square for categorical variables and ANOVA for continuous variables. Kaplan–Meier survival analysis was used for mortality and cumulative incidence analysis. Cox regression was used for thrombotic events and time to therapeutic aPTT. A value of *p* < 0.05 was considered to be significant. All data were analyzed using NCSS 2021 [14].

## Results

Overall, 22 patients with post-COVID-19 ARDS were treated with VV ECMO and placed on anticoagulation with the study agents during the analysis period. Out of the 22 patients, 10 patients received anticoagulation with UFH, and 12 patients received bivalirudin. Additional demographic characteristics can be found in Table 3.

**Table 3:** Baseline characteristics of patients (mean 95% or median IQR).

Characteristics	UFH (*n* = 10)	Bivalirudin (*n* = 12)	*p*-values
Age mean (y)	49.6 (42–57)	43.7 (38–49)	0.16
Weight (kg)	100 (81–119)	93.9 (86–102)	0.5
RACE, No. (%) *n* = 20			
White or Caucasian	0 (0)	4 (36)	0.03 Hispanic vs. white
Hispanic	9 (100)	6 (55)	0.6
African American	0 (0)	1 (8)	0.6
% Male	8 (80)	8 (67)	0.6
BMI mean (kg/m^2^)	34.3 (28–41)	31.7 (28–35)	NS
INR median (s)	1.3 (1.2–1.5)	1.3 (1.1–1.4)	0.6
aPTT baseline mean (s)	36.5 (33.2–39.7)	38.6 (31.2–46)	0.6
Hemoglobin median (g/dL)	11.3 (9.8–12.7)	10.2 (8.4–13.1)	0.14
Hematocrit median (%)	35.6 (31.6–39.6)	32.4 (26.8–38)	0.12
Platelet count median (×10^3^/μL)	281 (209–423)	184 (107–265)	0.052
AST median (IU/L)	29.5 (23–55)	26 (18–43)	NS
ALT median (IU/L)	76 (35.5–106)	30 (22–51)	0.09
SCr median (mg/dL)	0.84 (0.6–1.2)	0.54 (0.4–0.66)	0.04
Resp. rate (breaths/min)	28 (20–30)	30 (24–33)	NS
CRRT, No. (%)	6 (60)	1 (8)	0.02
Systolic BP mean (mmHg)	127 (115–139)	127 (110–144)	0.98
Diastolic BP mean (mmHg)	75 (65–85)	68 (58–79)	0.3
HR median (bpm)	95 (79–111)	90 (76–104)	0.6
Oxygen saturation median (%)	93 (84–97)	94.5 (87–97)	0.4
D-Dimer median (mcg/mL)	2.95 (1.7, 6.2)	2.35 (2.1–3.3)	0.3
Apache2 mean	14.5 (9.7–19.3)	10.4 (7.8–1.3)	0.12

BMI, body mass index; INR, international normalized ratio; aPTT, activated partial thromboplastin time; SCr, serum creatinine; CRRT, continuous renal replacement therapy; BP, blood pressure; HR, heart rate; NS, non-significant.

Mean time to therapeutic aPTT goal was 20 h for bivalirudin and 10 h for UFH (*p* = 0.02) (Table 4 / Figure 3). On average, patients on bivalirudin spent 50% of the time within their defined therapeutic aPTT goal range compared to 52% for the patients on UFH (Table 4).

**Table 4:** Primary endpoints.

	UFH (*n* = 10)	Bivalirudin (*n* = 12)	*p*-value (figure/test reference)
Time to therapeutic aPTT (hours)	9.9	19.9	0.02 (cumulative incidence analysis, Figure 2)
Time within therapeutic range (TTR %)	52	50	0.8
DVT, No. (%)	4 (40)	1 (8)	0.04
PE, No. (%)	4 (40)	2 (17)	
Thrombus in oxygenator, No. (%)	8 (80)	4 (33)	
Ischemic stroke, No. (%)	1 (10)	0 (0)	

DVT, deep vein thrombosis; PE, pulmonary embolism; aPTT, activated thromboplastin time.

Thrombotic events were documented in the bivalirudin group as 8% DVT, 17% PE, 33% oxygenator thrombus, and no patient suffered from ischemic stroke versus 40% DVT, 40% PE, 80% oxygenator thrombus, and 10% ischemic strokes in the UFH group (Table 4 / Figure 4). One patient in the bivalirudin arm and 2 patients in the UFH arm received tissue plasminogen factor (tPA) for the treatment of pulmonary embolism with cardiovascular deterioration (Table 5).

**Table 5:** Hospital interventions.

	UFH (*n* = 10)	Bivalirudin (*n* = 12)	*p*-value (test reference)
tPA use^a^ (*n*, %)	2 (20)	1 (8)	–
Hemoglobin drop^b^ (*n*, %)	10 (100)	7 (41)	0.04
Platelets transfused	10 (100)	8 (67)	–
Cryoprecipitate transfused	7(70)	8 (67)	–
Bleeding requiring surgery	2 (20)	0 (0)	0.2
Hemorrhagic stroke (*n*, %)	3 (30)	0 (0)	0.08
Time on ECMO median (days) *n* = 20	49	29.5	0.8
Median dose	12.5 units/kg/h	0.15 mg/kg/h	–
Loading dose	2 (20)	1 (8)	–

tPA, tissue plasminogen factor.

a For the treatment of pulmonary embolism with cardiovascular deterioration.

b Hemoglobin drop defined as at least 2 g/dL in a 24-h period.

Overall bleeding was higher in patients treated with UFH (90%) than bivalirudin (75%). Significant bleeding requiring surgical intervention occurred in two patients (20%) in the UFH. No patient in the bivalirudin arm required surgery for the occurrence of bleeding events (Table 6). Hemorrhagic stroke occurred in three patients (30%) in the UFH group (Table 5). Out of the three patients with hemorrhagic stroke, only one patient had received tPA for the treatment of PE. All patients (100%) receiving heparin experienced a hemoglobin drop of at least 2 g/dL in 24-h period or greater than 20 mL/kg over a 24-h period, compared to seven patients (41%) receiving bivalirudin. The most commonly documented minor bleeding event was epistaxis and the most frequent major bleeding event was blood loss from chest tubes and ECMO cannulation sites. Five patients on UFH required anticoagulation to be held and a reduction of the aPTT target range versus 3 patients in the bivalirudin group.

**Table 6:** Secondary endpoints.

	UFH (*n* = 10)	Bivalirudin (*n* = 12)	*p*-value (figure reference)
Overall bleeding (%)	9 (90)	9 (75)	0.6
Bleeding requiring surgical intervention (%)	2 (20)	0	0.2
Hemorrhagic stroke (%)	3 (30)	0	0.08
LOS (median, days)	65	119	0.5 (Figure 1)
LOS 90 day (median, days)	40	40	0.4 (Figure 2)
Mortality (%)	9 (90)	7 (58)	0.5 (Figure 1)
Mortality 90 day (%)	7 (70)	5 (42)	0.2

LOS, length of stay.

**Figure 1:**
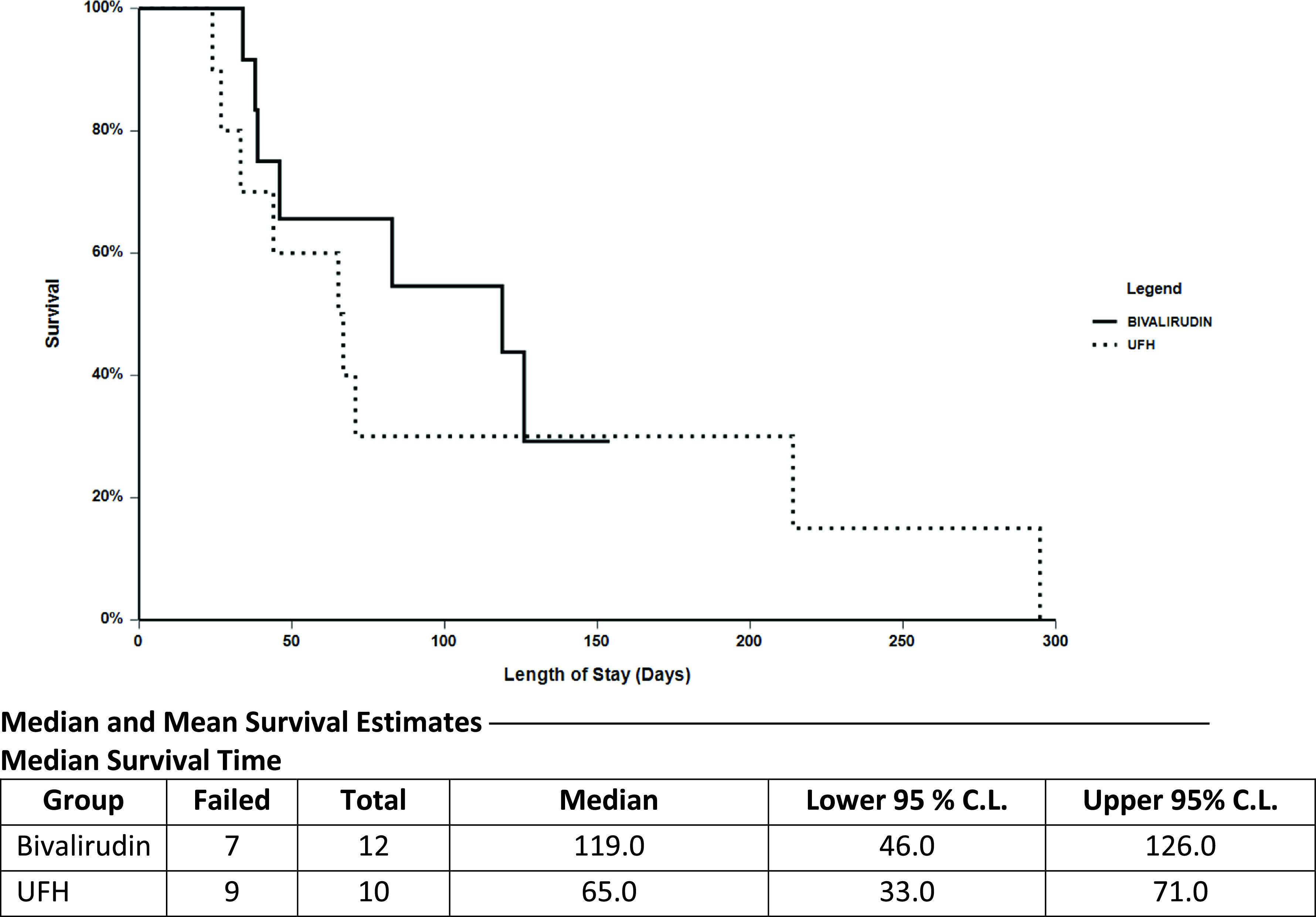
Survival analysis (unrestricted).

The median time spent on ECMO support was 29 days in the bivalirudin arm and 49 days in the UFH arm (*p* = 0.8) (Table 6). Death occurred in seven patients (58%) during their admission in the bivalirudin group and nine patients (90%) in the UFH group (Table 6 / Figure 1). All reported deaths were either due to patient expiration while on VV ECMO support or patients were weaned from VV ECMO and all supportive care due to terminal state.

**Figure 2:**
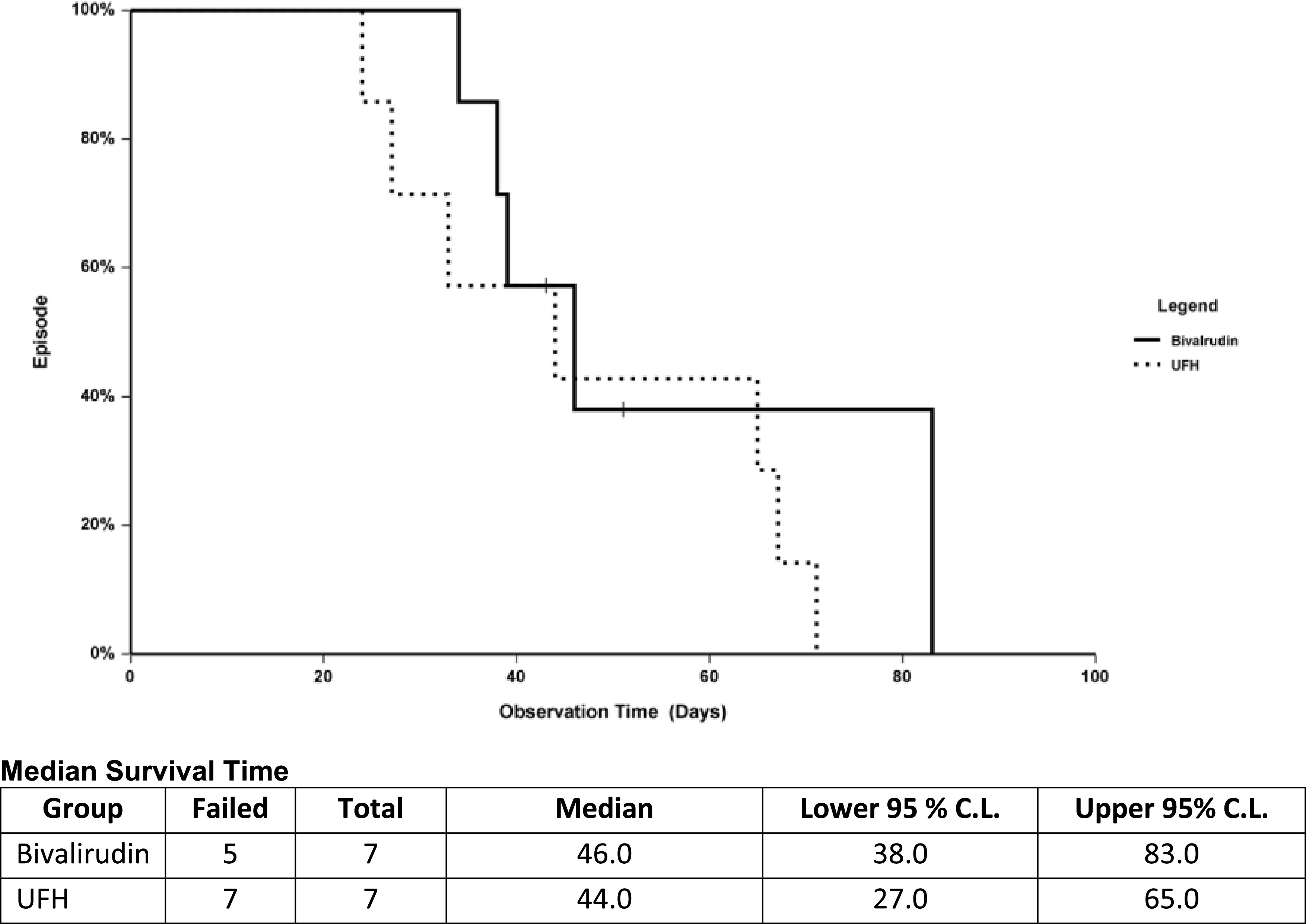
Survival analysis 90 day.

**Figure 3:**
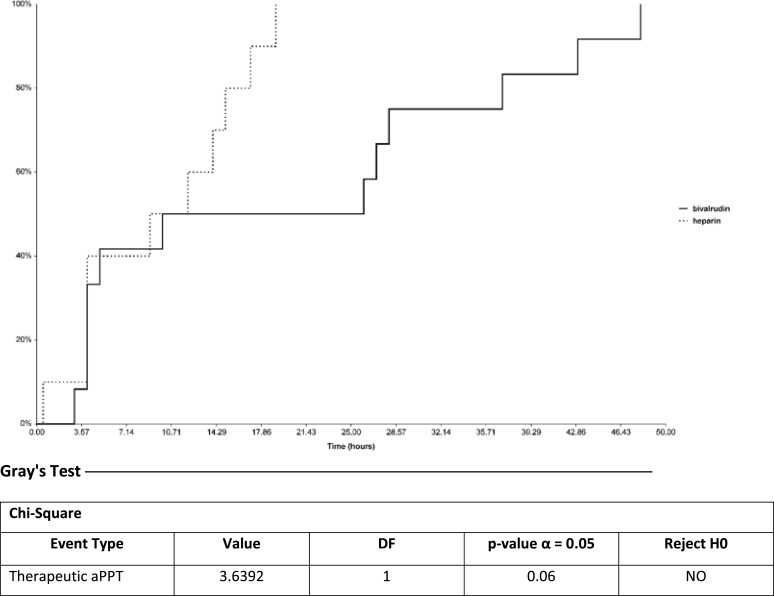
Cumulative incidence analysis for time to therapeutic aPTT.

**Figure 4:**
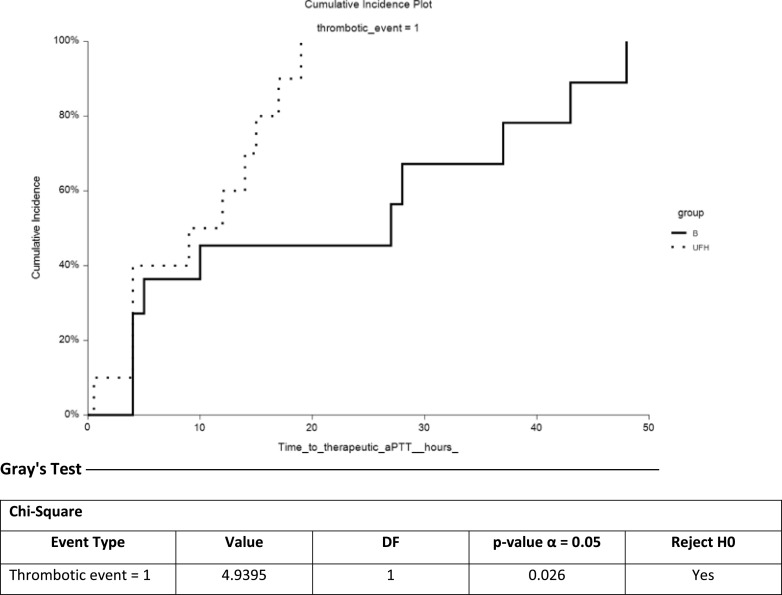
Cumulative incidence analysis for time to thrombotic events.

## Discussion

The use of ECMO has become a more frequent rescue therapy for patients with ARDS after the COVID-19 pandemic started in 2019. One of the clinical challenges to supporting this patient population with VV ECMO is anticoagulation, especially in the setting of SARS-CoV-2 pneumonia due to its association with an increased prothrombotic state and complications for COVID-19-positive patients [15]. Although adequate anticoagulation is more critical during VA ECMO compared with VV ECMO therapy to reduce the risk of stroke, the hypercoagulable pathophysiology of SARS-CoV-2 makes it also vital in patients supported with the venovenous modality.

UFH has been the most used anticoagulant for ECMO due to its rapid onset of action, availability, cost-effectiveness, long history of use, and ease of reversal with protamine. However, UFH carries its own set of limitations that could predispose patients to fluctuations in dose sensitivity and heparin-induced thrombocytopenia (HIT) [16]. As previously mentioned, bivalirudin offers potential advantages during ECMO due to its ability to attach directly to and inhibit both free circulating and fibrin-bound thrombin. Furthermore, bivalirudin’s short half-life and duration of action allows for rapid achievement of steady state, titration to target goal anticoagulation, and halting of anticoagulant effects when necessary. Due to the current study’s limitations, including the absence of an established protocol for anticoagulation with bivalirudin as well as a less aggressive approach to reach aPTT targets with bivalirudin dosing and subsequent adjustments, it was not possible to show the advantage of the DTI in achieving a rapid steady state. An additional advantage to the use of bivalirudin is the lack of a structural relationship between the DTI and heparin, therefore, avoiding the risk of HIT [17].

A growing body of evidence has shown the use of bivalirudin to be associated with decreased circuit-related thrombotic events and blood product transfusion [18] as well as lower bleeding and thrombotic events [11–13]. Rivosecchi *et al*. evaluated anticoagulation with bivalirudin and UFH in 2021 by assessing the presence of ECMO in-circuit-related thrombotic complications and the volume of blood products administered during extracorporeal membrane oxygenation duration. The study concluded that patients receiving bivalirudin for systemic anticoagulation on VV ECMO experienced a decrease in the number of ECMO circuit-related thrombotic events as well as a significant decrease in the volume of blood products administered [18]. Later in 2022, Bissell *et al*. confirmed that bivalirudin was a viable choice for anticoagulation in patients on ECMO for severe respiratory failure secondary to COVID-19, showing lower rates of bleeding at 15.1%, and only 6.1% of patients developing a new venous thromboembolic event while on ECMO [13]. Previous trials in 2021 [11, 12] had already shed light on the role of bivalirudin in COVID-19 by offering a potential option for maintaining systemic anticoagulation during ECMO in a manner that mitigates the prothrombotic nature of the underlying pathophysiologic state and maintaining more consistently aPTT goals in this population. Despite the above-published studies, the existing literature comparing bivalirudin versus UFH in VV ECMO supporting COVID-19-positive patients remains limited with the need for more trials to support the efficacy and safety of bivalirudin for anticoagulation of COVID-19 patients on ECMO.

The enhanced pharmacokinetic profile of bivalirudin with a strong correlation between its dose and anticoagulant effects was observed in the current study. In our analysis patients on bivalirudin remained within their defined therapeutic aPTT target range (TTR %) very similarly to patients in the UFH group with lower incidence of thrombotic events. Thrombus in the oxygenator, ischemic strokes, and overall thrombotic events (DVT/PE) were significantly lower in the bivalirudin group (*p* = 0.04) (Table 3). Overall bleeding was also lower in the bivalirudin arm with no patients suffering from hemorrhagic strokes or requiring surgical intervention to control major bleeding events (Table 4.). While not proven to be statistically significant (*p* = 0.5), the mortality rate was 58% in patients receiving bivalirudin versus 90% in patients receiving UFH (Figure 1 / Table 4). No patient experienced HIT in either group of the study and the length of stay was similar for both arms.

### Study limitations

Limitations of this study include a single-center approach, its retrospective design, small population size, and reliance on EHR documentation for data collection. During the time period retrospectively analyzed, the hospital had an established UFH protocol for dosing and monitoring but not an approved protocol for anticoagulation with bivalirudin. The latter fact might have impacted the study results regarding the time to reach therapeutic aPTT goals and initial bleeding incidence since a less aggressive approach to reach aPTT targets was assumed with bivalirudin dosing and subsequent adjustments.

Substantial progress had been made in COVID-19-specific therapies since the beginning of the pandemic in 2020, which might have impacted patient outcomes in the current study. Patient data in the UFH group were analyzed for the period of 2020 while bivalirudin patient data was collected in 2021. As per the APACHE data reflected in Table 1, patients in the UFH group had more severe disease than patients in the bivalirudin group as well as fewer available specific therapies for the treatment of COVID-19 (*p* = 0.12). Physician-directed aPTT goals and periods of time where anticoagulation was held due to medical procedures (i.e., bronchoscopy, chest tube insertion/removal, etc.) could have also impacted the internal validity of the outcomes by adding inconsistency to the time patients remained in therapeutic aPTT.

### Study conclusions

Despite these facts and to the best of our knowledge, this study has a relatively large sample size in comparison to the existent literature analyzing bivalirudin versus UFH in COVID-19-positive patients with ARDS receiving VV ECMO support. In hospitalized patients with COVID-19-associated ARDS on VV ECMO support, the use of bivalirudin showed to be a viable anticoagulation alternative in terms of efficacy compared to UFH and resulted in a favorable safety profile with lower rates of bleeding and thrombotic events. Future randomized trials are needed to further corroborate the efficacy and safety of bivalirudin in a larger population of patients receiving VV ECMO support for COVID-19-related ARDS.

## Data Availability

The research data associated with this article are included in the article.
